# Room temperature deformation of single crystals of Ti_5_Si_3_ with the hexagonal D8_8_ structure investigated by micropillar compression tests

**DOI:** 10.1038/s41598-020-75007-7

**Published:** 2020-10-22

**Authors:** Kyosuke Kishida, Takayoshi Fukuyama, Takuto Maruyama, Haruyuki Inui

**Affiliations:** 1 Department of Materials Science and Engineering, Kyoto University, Sakyo-ku, Kyoto, 606-8501 Japan; 2 Center for Elements Strategy Initiative for Structural Materials (ESISM), Kyoto University, Sakyo-ku, Kyoto, 606-8501 Japan

**Keywords:** Materials science, Structural materials, Mechanical properties, Metals and alloys

## Abstract

Micropillar compression tests of Ti_5_Si_3_ single crystals were conducted at room temperature as a function of loading axis orientation and specimen size in order to investigate their room temperature plastic deformation behavior. Plastic flow by the operation of three deformation modes, {1$${\overline{1}}$$00}[0001], {2$${\overline{1}}$$$${\overline{1}}$$2} < 2$${\overline{1}}$$$${\overline{1}}$$$${\overline{3}}$$ > and {1$${\overline{1}}$$01} < 2$${\overline{1}}$$$${\overline{1}}$$$${\overline{3}}$$ > slip were observed in [2$${\overline{2}}$$05]-, [0001]- and [4$${\overline{3}}$$$${\overline{1}}$$0]-oriented micropillar specimens deformed at room temperature, respectively. The CRSS values were evaluated to be very high above 2.7 GPa and were confirmed to increase up to about 6 GPa with the decrease in the specimen size. The fracture toughness values are evaluated to be 0.45 MPa m^1/2^ (notch plane // (0001)) and 0.73 MPa m^1/2^ (notch plane //(1$${\overline{1}}$$00)) based on the results of micro-cantilever bend tests of chevron-notched specimens. The fracture toughness values are considerably lower than those for D8_*l*_-Mo_5_SiB_2_ and D8_*l*_-Nb_5_Si_3_ evaluated by the same method, indicating the inherent brittleness of binary Ti_5_Si_3_ compared to the other transition-metal silicides of the TM_5_Si_3_ type (TM: transition-metal).

## Introduction

Since the gas inlet temperature of the advanced turbine systems already exceeds 1700 °C that is much higher than the melting temperature (~ 1350 °C) of Ni-based superalloys, there is an ever-increasing demand for developing ultra high-temperature structural materials that can withstand at temperatures well above the maximum operating temperatures of Ni-base superalloys in severe oxidizing atmosphere^1,2^. Silicides with the chemical formula of TM_5_Si_3_ formed with transition-metals (TMs) are of interest, as most of them exhibit the highest melting temperature in each of the corresponding TM-Si binary phase diagrams and a reasonably good oxidation property due to the high Si content^3^. Indeed, Nb_5_Si_3_ and Mo_5_SiB_2_ with the tetragonal D8_*l*_ structure and Mo_5_Si_3_ with the tetragonal D8_m_ structure have intensively been investigated in the last several decades as the strengthening phases of Nb-^4–7^ and Mo-^8–10^ based refractory alloys, respectively. Ti_5_Si_3_ with the hexagonal D8_8_ structure, on the other hand, has been investigated initially as a constituent phase of Ti-Ti_5_Si_3_ in-situ composites^11^ and in recent years as the strengthening phase of Mo-Mo_5_SiB_2_ based alloys because the creep strength and oxidation resistance are significantly improved by replacing a large amount of Mo with Ti so as to incorporate Ti_5_Si_3_ as the constituent phase of Mo-Mo_5_SiB_2_ based alloys^12–17^. Because of some excellent properties as a promising structural material for ultra-high temperature applications [the high melting temperature (2130 °C) and low density (4.32 g/cm^3^)], the deformation behavior of monolithic Ti_5_Si_3_ has been investigated with polycrystals^11^ and single crystals^18,19^. Frommeyer et al.^11^ reported that Ti_5_Si_3_ is extremely brittle at low temperatures as expected from the complex crystal structure of the D8_8_ type (Pearson symbol: *hP*16, Space group: *P*6_3_/*mcm*). Plastic flow is observed only above 1000 °C in polycrystals, while Umakoshi and Nakashima^18^ reported plastic flow carried by deformation twinning of the {1$$\overline{1}$$02} < $$\overline{1}$$101 > -type at high temperatures above 1300 °C in single crystals with some limited orientations. Then, Kishida et al.^19^ made a first systematic deformation experiment on single crystals of Ti_5_Si_3_ in uniaxial compression and found that plastic flow is possible only above 1200 °C regardless of crystal orientation with three different operative deformation modes, {1$$\overline{1}$$00} < 0001 > prismatic slip, {2$$\overline{1}$$$$\overline{1}$$2} < 2$$\overline{1}$$$$\overline{1}$$$$\overline{3}$$ > pyramidal slip and {2$$\overline{1}$$$$\overline{1}$$8} < 8$$\overline{4}$$$$\overline{4}$$$$\overline{3}$$ > twinning depending on crystal orientation.

Recently, micropillar compression testing that utilizes small specimens of the micron-meter scale has been recognized as a powerful method to investigate the plastic deformation behavior of hard and brittle materials at a low temperature (for example, room temperature) well below their onset temperatures (above 1000 °C for most of these materials) for plastic flow in the bulk form. Those hard and brittle materials include intermetallics with complex crystal structures^20–25^, semiconductors^26–31^ and ceramics^32–35^. We have investigated the room-temperature plastic deformation behavior of various hard and brittle materials including D8_*l*_-Mo_5_SiB_2_^24^, D8_*l*_-Nb_5_Si_3_^25^, 6*H*-SiC^31^ and so on^21–23^ and have successfully identified operative slip systems and their critical resolved shear stress (CRSS). Of particular interest to note is that slip systems that do not participate in the high temperature deformation in the bulk form do participate in the room-temperature deformation in the micropillar form^24,25,31^ and that completely different dislocation dissociation schemes are observed at high (bulk) and low (micropillar) temperatures even when the same slip system operates. Thus, the CRSS at a low temperature obtained in the micropillar form is plotted far away from the extrapolation of the temperature-dependent CRSSs obtained at high temperatures in the bulk form^31^. Such information about the room-temperature deformation behavior must be very important to conceive a strategy to improve the brittleness at low temperatures of these hard and brittle materials.

In the present study, we investigate the room-temperature deformation behavior of single crystals of Ti_5_Si_3_ with the hexagonal D8_8_ structure as a function of specimen size and loading-axis orientation by micropillar compression tests, to get information that might be useful for the improvement of the brittleness at low temperatures.

## Results

### Compression deformation behavior

**Figure 1:**
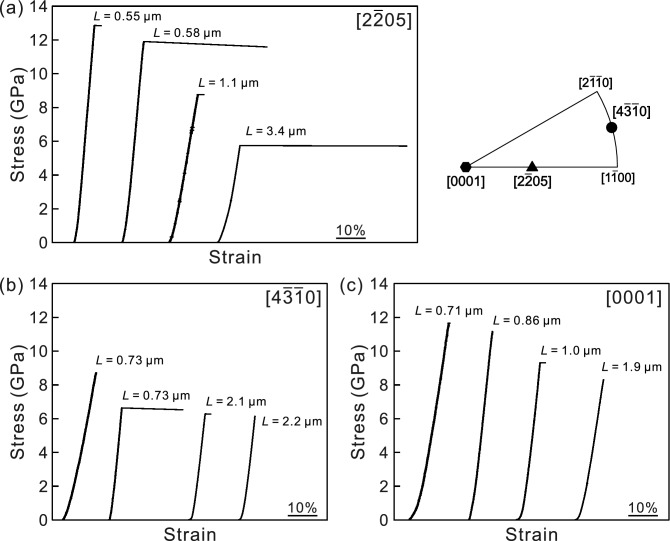
Typical stress–strain curves obtained for micropillar specimens of Ti_5_Si_3_ single crystals with the loading-axis orientations of (**a**) [2$$\overline{2}$$05], (**b**) [4$$\overline{3}$$$$\overline{1}$$0] and (**c**) [0001].

Figure 1a–c show typical stress–strain curves obtained for single crystals of micropillar specimens with the [2$$\overline{2}$$05], [4$$\overline{3}$$$$\overline{1}$$0] and [0001] orientations, respectively. A relatively large flat portion corresponding to a strain burst is commonly observed soon after elastic loading in many stress–strain curves regardless of the loading-axis orientation. Although a strain burst is followed by a load drop in most cases, that is not included in Fig. 1. As observed in other micropillar compression tests of single crystals of metallic materials^36–40^, the occurrence of a strain burst in stress–strain curves in Ti_5_Si_3_ is considered to be plastic flow caused by dislocation motion in avalanches, as in the case of Mo_5_SiB_2_^24,37,40^.

**Figure 2:**
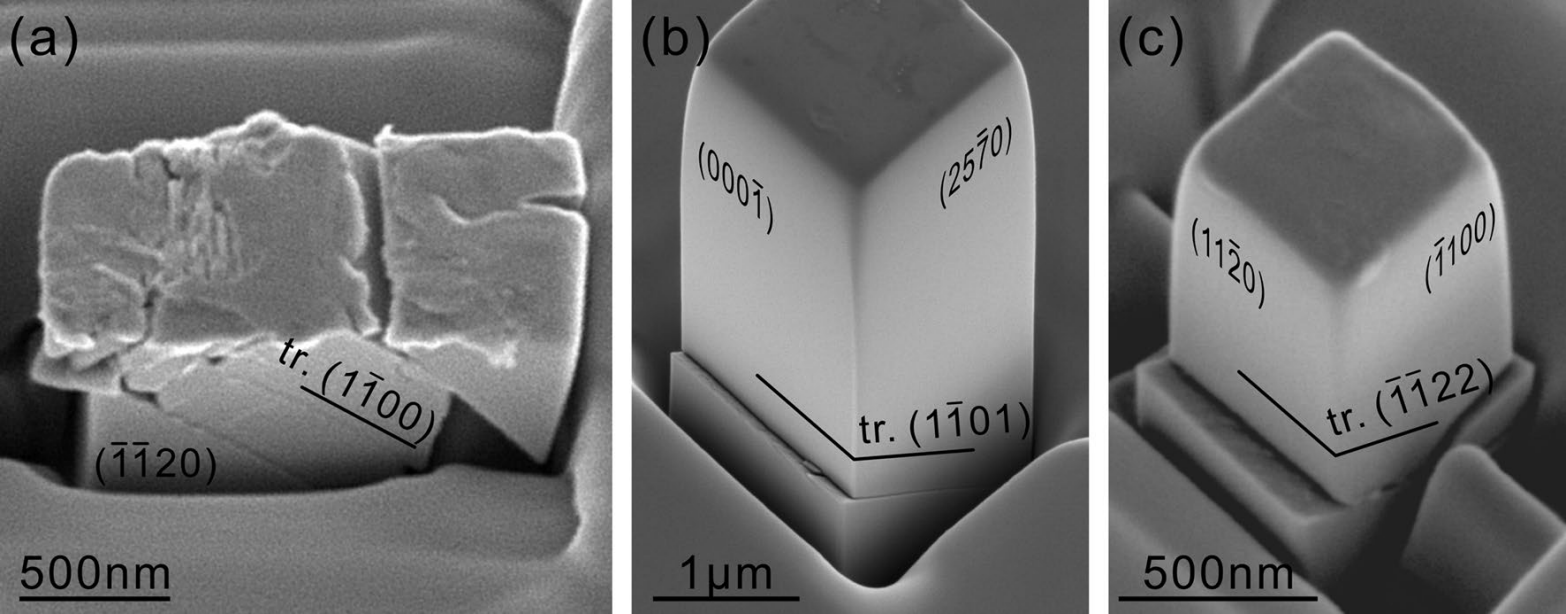
Scanning electron microscopy images of Ti_5_Si_3_ single-crystalline micropillar specimens with (**a**) [2$$\overline{2}$$05], (**b**) [4$$\overline{3}$$$$\overline{1}$$0] and (**c**) [0001] orientations after compression.

Being consistent with the occurrence of plastic flow caused by dislocation motion in avalanches, most micropillar specimens exhibit slip plane (shear) failure, accompanied by giant steps on the slip plane along the slip direction (Fig. 2). Deformation microstructures with the three orientations are shown in Fig. 2a–c for some specimens in which micropillar testing was successfully interrupted before instantaneous slip plane failure occurs. For the [2$$\overline{2}$$05] orientation (Fig. 2a), analysis of slip traces that appeared on two orthogonal side-surfaces has identified the slip plane to be (1$$\overline{1}$$00). Because shear deformation occurs so as to maintain the ($$\overline{1}\overline{1}$$20) side-surface flat, the shear direction is contained in the ($$\overline{1}$$$$\overline{1}$$20) plane and is determined to be [0001]. The slip system operative in [2$$\overline{2}$$05]-oriented micropillar specimens is thus identified to be (1$$\overline{1}$$00)[0001], as observed in the same orientation of bulk single crystals at above 1300 °C^19^.

For the [4$$\overline{3}$$$$\overline{1}$$0] orientation, deformation markings corresponding to slip on (1$$\overline{1}$$01) are observed on two orthogonal side faces. On the assumption that slip on this plane occurs along either **a** or **a** + **c** direction (when expressed with the hexagonal lattice parameters), the possible slip vectors in the [4$$\overline{3}$$$$\overline{1}$$0] orientation are [11$$\overline{2}$$0], [2$$\overline{1}$$$$\overline{1}$$$$\overline{3}$$] and [1$$\overline{2}$$1$$\overline{3}$$], the latter two of which have the identical Schmid factors. Of the three, [11$$\overline{2}$$0] is ruled out, since deformation markings are clearly observed also on the (000$$\overline{1}$$) side-surface that contains the [11$$\overline{2}$$0] direction (Fig. 2b). The slip system identified in [4$$\overline{3}$$$$\overline{1}$$0]-oriented micropillar specimens is thus identified to be {1$$\overline{1}$$01} < 2$$\overline{1}$$$$\overline{1}$$$$\overline{3}$$ > . This slip system has never been identified to operate in bulk single crystals. In bulk single crystals with the same [4$$\overline{3}$$$$\overline{1}$$0] orientation, {2$$\overline{1}$$$$\overline{1}$$2} < 2$$\overline{1}$$$$\overline{1}$$$$\overline{3}$$ > pyramidal slip was identified to operate above 1400 °C^19^.

For the [0001] orientation, slip trace analysis reveals that slip occurs on the (11$$\overline{2}$$2) slip plane. Because 1/3 < $$\overline{1}$$$$\overline{1}$$2$$\overline{3}$$ > is the shortest lattice translation vector (0.906 nm) on {11$$\overline{2}$$2} slip planes, the operative slip system in [0001]-oriented micropillar specimens is inferred to be {2$$\overline{1}$$$$\overline{1}$$2} < 2$$\overline{1}$$$$\overline{1}$$$$\overline{3}$$ > . As described above, {2$$\overline{1}$$$$\overline{1}$$2} < 2$$\overline{1}$$$$\overline{1}$$$$\overline{3}$$ > slip has been observed in [4$$\overline{3}$$$$\overline{1}$$0]-oriented bulk single crystals above 1400 °C but not in [0001]-oriented bulk single crystals, in which deformation twinning of the {2$$\overline{1}$$$$\overline{1}$$8} < 8$$\overline{4}$$$$\overline{4}$$$$\overline{3}$$ > -type is operative above 1400 °C^19^.

### Fracture toughness

**Figure 3:**
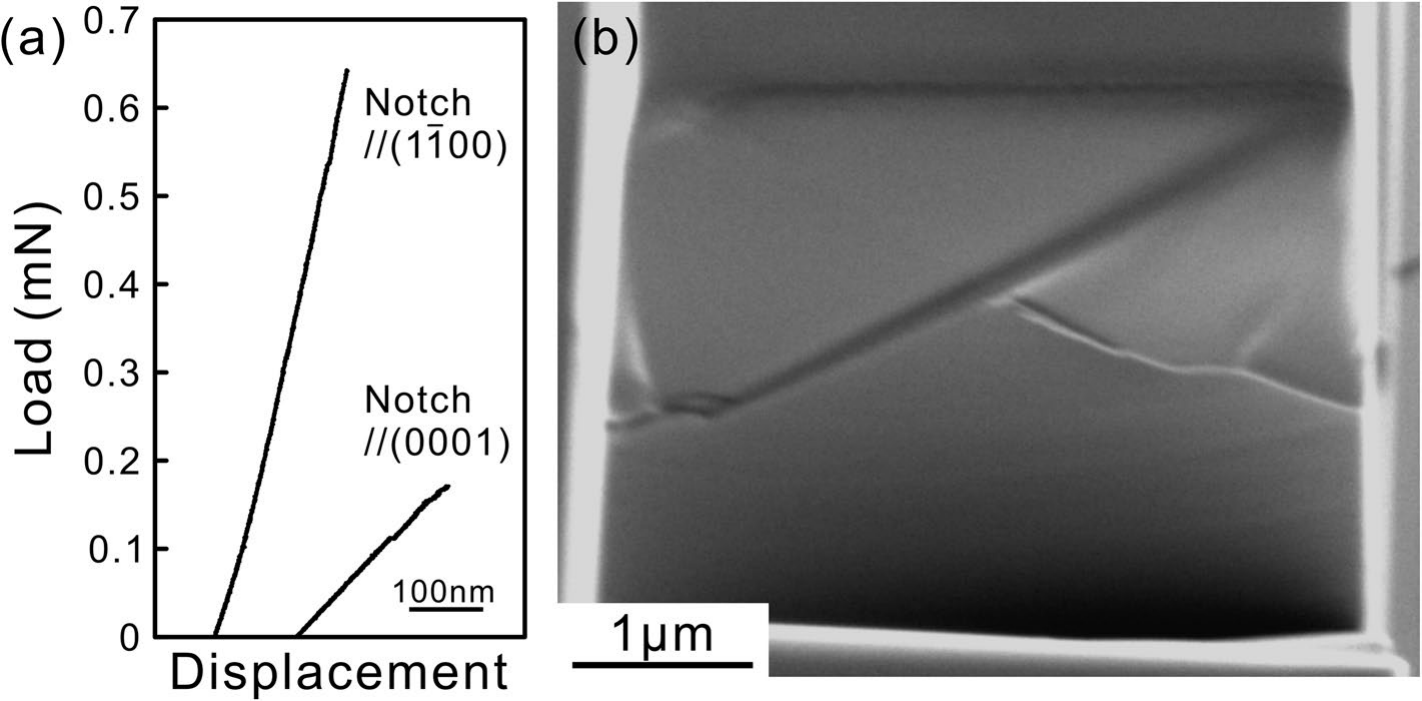
(**a**) Load–displacement curves obtained in micro-cantilever bend tests of chevron-notched micro-beam specimens with a notch plane parallel to either (0001) or (1$\overline{1}$00). (**b**) The fracture surface of the fractured micro-beam specimen with a notch plane parallel to (0001).

Micro-cantilever bend tests on chevron-notched micro-beam specimens were conducted to evaluate the fracture toughness. The load–displacement curves obtained for chevron-notched micro-beam specimens with a notch plane parallel to (0001) and (1$\overline{1}$00) are shown in Fig. 3a. The loading directions were set parallel to [1$\overline{1}$00] and [0001] for (0001)- and (1$\overline{1}$00)-notched micro-beam specimens, respectively. The load–displacement curves (Fig. 3a) exhibit no sign of apparent plastic deformation before failure occurs. In addition, a fairly flat fracture surface observed in the SEM image for the (0001)-notched micro-beam specimen indicates the occurrence of brittle cleavage fracture (Fig. 3b). The values of fracture toughness can then be evaluated simply from the maximum load and geometrical parameters of the chevron-notched specimens based on the model proposed by Deng et al.^41^, details of which were described in our previous papers^24^. The fracture toughness values of Ti_5_Si_3_ are evaluated to be 0.45 and 0.73 MPa m^1/2^ respectively with the notch plane parallel to (0001) and (1$\overline{1}$00), both of which are considerably lower than those for D8_*l*_-Mo_5_SiB_2_ (2.43 MPa m^1/2^; notch plane // (100)) and D8_*l*_-Nb_5_Si_3_ (1.79 MPa m^1/2^; notch plane // (001)) evaluated by the same method^24,25^.

## Discussion

**Figure 4:**
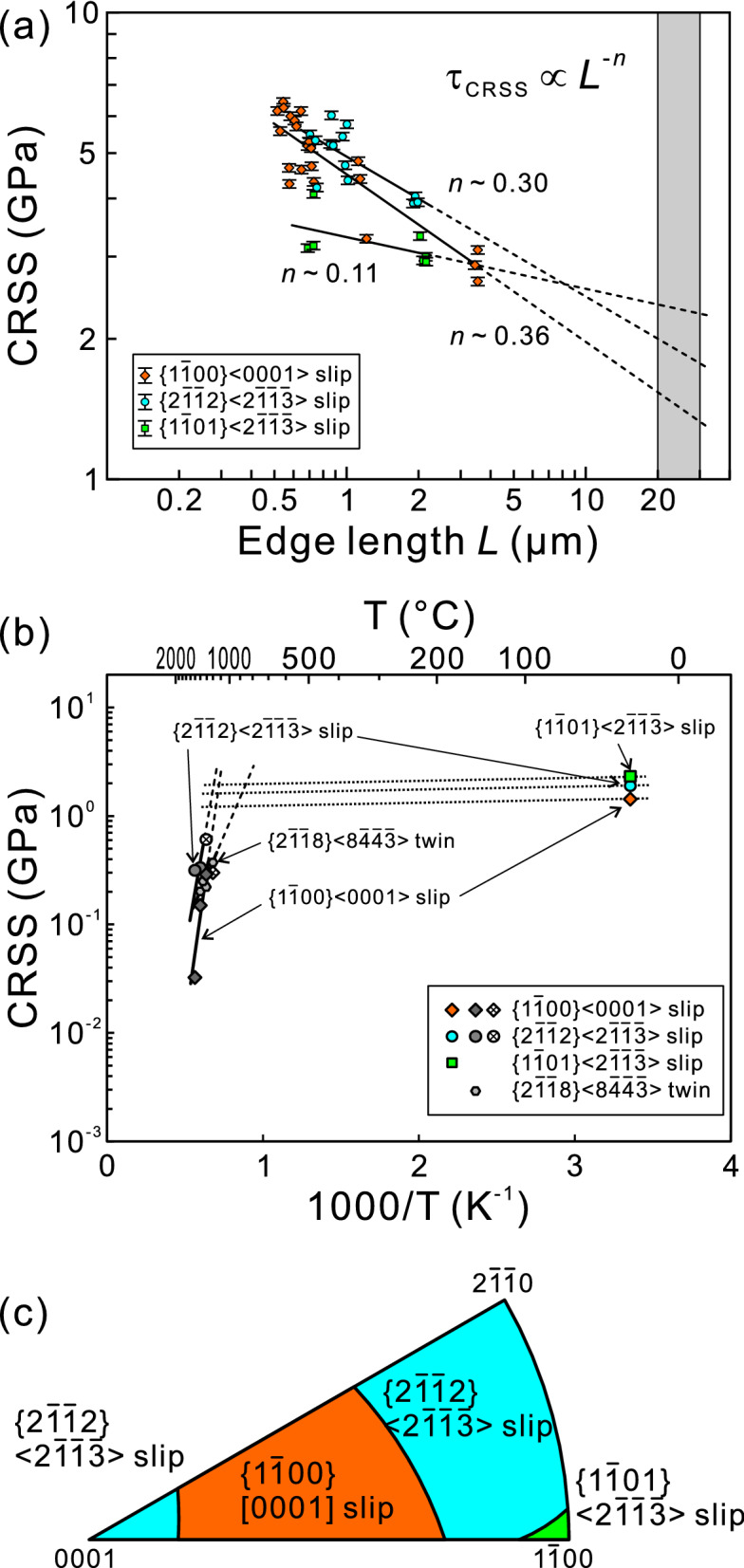
(**a**) Specimen size dependence of CRSS for {1$$\overline{1}$$00}[0001], {2$$\overline{1}$$$$\overline{1}$$2} < 2$$\overline{1}$$$$\overline{1}$$$$\overline{3}$$ > and {1$$\overline{1}$$01} < 2$$\overline{1}$$$$\overline{1}$$$$\overline{3}$$ > slip. Vertical error bars correspond to the possible 2% stress variation caused by errors in measurements of specimen dimensions. (**b**) Comparison of estimated bulk CRSS values at room temperature with those at high temperatures reported in^19^. (**c**) Orientation dependence of the operative slip systems under uniaxial compression calculated with the estimated bulk CRSS values for the three identified slip systems.

The CRSS values (τ_CRSS_) for the three slip systems identified in the present study are calculated with yield stresses defined as the stress at which the first strain burst occurs and the corresponding Schimid factors as plotted in Fig. 4a as a function of specimen size (the edge length *L*). The CRSS values for all slip systems are extremely high exceeding 2.6 GPa regardless of specimen size and exhibit the so-called “smaller is stronger” trends, following an inverse power-law relationship, τ_CRSS_ ∝ *L*^−*n*^ (*n*: the power-law exponent), as commonly observed in micropillar compression of single crystals of many metallic materials, ceramics, semiconductors and intermetallic compounds^22–25,30–33,35,36,38,39,42,43^. The power-law exponents for the three slip systems of {1$$\overline{1}$$00}[0001], {2$$\overline{1}$$$$\overline{1}$$2} < 2$$\overline{1}$$$$\overline{1}$$$$\overline{3}$$ > and {1$$\overline{1}$$01} < 2$$\overline{1}$$$$\overline{1}$$$$\overline{3}$$ > are estimated to be 0.36, 0.30 and 0.11, respectively. The power-law exponents in hard and brittle materials such as 6*H*-SiC (*n* = 0.10–0.21), Mo_5_SiB_2_ (*n* = 0.16–0.22) are generally much lower than those reported for face-centered cubic (FCC) metals (0.5–1.0) and body-centered cubic (BCC) metals (0.2–0.5)^38,39,42^. However, the power-law exponents observed for the two slip systems (*n* = 0.36 and 0.30 for {1$$\overline{1}$$00}[0001] and {2$$\overline{1}$$$$\overline{1}$$2} < 2$$\overline{1}$$$$\overline{1}$$$$\overline{3}$$ > slip) in Ti_5_Si_3_ are not as low as those for 6*H*-SiC and Mo_5_SiB_2_ but are comparably high as those observed for some particular slip systems (0.43 and 0.38 for (001) < 010 > and {110} < 1$$\overline{1}$$0 > slip) in α-Nb_5_Si_3_^25^. In micropillar compression experiments of single crystals of FCC and BCC metals, the inverse power-low curve of size-dependent CRSS is known to coincide with the bulk CRSS when the specimen size is in the range of 20–30 μm^43^. If we assume that the same criteria is applicable to Ti_5_Si_3_, the bulk CRSS values for {1$$\overline{1}$$00}[0001], {2$$\overline{1}$$$$\overline{1}$$2} < 2$$\overline{1}$$$$\overline{1}$$$$\overline{3}$$ > and {1$$\overline{1}$$01} < 2$$\overline{1}$$$$\overline{1}$$$$\overline{3}$$ > are estimated as the first approximation respectively to be 1.43 ± 0.10, 1.90 ± 0.10 and, 2.32 ± 0.06 GPa, from the extrapolation of the inverse power-low curves.

Figure 4b compares the estimated bulk CRSS values at room temperature for the three slip systems, {1$$\overline{1}$$00}[0001], {2$$\overline{1}$$$$\overline{1}$$2} < 2$$\overline{1}$$$$\overline{1}$$$$\overline{3}$$ > , and {1$$\overline{1}$$01} < 2$$\overline{1}$$$$\overline{1}$$$$\overline{3}$$ > with those obtained in high-temperature compression experiments of bulk single crystals. In bulk compression experiments, we have identified three operative deformation modes, {1$$\overline{1}$$00}[0001] prism slip, {2$$\overline{1}$$$$\overline{1}$$2} < 2$$\overline{1}$$$$\overline{1}$$$$\overline{3}$$ > pyramidal slip and {2$$\overline{1}$$$$\overline{1}$$8} < 8$$\overline{4}$$$$\overline{4}$$$$\overline{3}$$ > twinning and have confirmed that the CRSS for these three deformation modes strongly depend on temperature. For a constant strain-rate experiments, a linear correlation is generally observed between ln τ_CRSS_ and (1/*T*), since the strain rate $$\dot{\gamma }$$ can be expressed as a function of CRSS, temperature *T* and activation energy *Q* for thermal activation process for dislocation glide with the following equation.

1 $$ \dot{\gamma } \propto \tau_{{{\text{CRSS}}}}^{m} \exp ( - Q/kT) $$

where *m* is the stress exponent and *k* is the Boltzmann constant^44^. Apparently, the room-temperature bulk CRSS values estimated for {1$$\overline{1}$$00}[0001] prism slip, {2$$\overline{1}$$$$\overline{1}$$2} < 2$$\overline{1}$$$$\overline{1}$$$$\overline{3}$$ > pyramidal slip are much lower than those expected from the extrapolation of the ln τ_CRSS_ − (1/T) relations for the deformation modes operative at high temperatures. This suggests that the deformation mechanism that governs the CRSS for {1$$\overline{1}$$00}[0001] prism slip and {2$$\overline{1}$$$$\overline{1}$$2} < 2$$\overline{1}$$$$\overline{1}$$$$\overline{3}$$ > pyramidal slip varies depending on testing temperature. One possible explanation for the different deformation mechanisms operating at high and low temperatures is that the room-temperature bulk CRSSs estimated in the micropillar form is determined by the nucleation of new dislocations from specimen surfaces of micropillars, as we discussed previously for 6*H*-SiC^31^ and α-Nb_5_Si_3_^25^ because virtually no grown-in dislocation is expected to exist in micropillar specimens of Ti_5_Si_3_ single crystals when judged from the brittleness and high CRSSs. However, more detailed studies including dislocation analysis by TEM are definitely needed to prove this.

The expected orientation-dependent operative slip systems are calculated based on the estimated bulk CRSS values for the three slip systems identified in micropillar compression tests and are shown in the stereographic projection of Fig. 4c. As expected from the relatively low CRSS values for {1$$\overline{1}$$00}[0001] and {2$$\overline{1}$$$$\overline{1}$$2} < 2$$\overline{1}$$$$\overline{1}$$$$\overline{3}$$ > slip, the orientation range for these two slip systems to operate are very wide, while that for {1$$\overline{1}$$01} < 2$$\overline{1}\overline{1}$$$$\overline{3}$$ > slip is quite limited to near [1$$\overline{1}$$00]. A sufficient number of independent slip systems is achieved for general deformation of polycrystalline aggregates by the combination of {1$$\overline{1}$$00}[0001] and {2$$\overline{1}$$$$\overline{1}$$2} < 2$$\overline{1}$$$$\overline{1}$$$$\overline{3}$$ > slip, both of which have a relatively smaller CRSS values (1.43 ± 0.10 and 1.90 ± 0.10 MPa) when compared to {1$$\overline{1}$$01} < 2$$\overline{1}$$$$\overline{1}$$$$\overline{3}$$ > slip (2.32 ± 0.06 GPa).

For the [0001] orientation, {2$$\overline{1}$$$$\overline{1}$$2} < 2$$\overline{1}$$$$\overline{1}$$$$\overline{3}$$ > pyramidal slip is found to operate in micropillar specimens at room temperature, while {2$$\overline{1}$$$$\overline{1}$$8} < 8$$\overline{4}$$$$\overline{4}$$$$\overline{3}$$ > twinning is operative in bulk single crystals at high temperatures above 1300°C^19^. The absence of {2$$\overline{1}$$$$\overline{1}$$8} < 8$$\overline{4}$$$$\overline{4}$$$$\overline{3}$$ > twinning at room temperature is easily understood from the fact that {2$$\overline{1}$$$$\overline{1}$$8} < 8$$\overline{4}$$$$\overline{4}$$$$\overline{3}$$ > twinning requires very complicated atomic shuffling to restore the crystal structure during deformation twinning, so that it can happen only at sufficiently high temperatures at which sufficient atomic diffusion occurs^19^. That must be the reason why the [0001]-oriented micropillar specimens need to find out alternative slip system, {2$$\overline{1}$$$$\overline{1}$$2} < 2$$\overline{1}$$$$\overline{1}$$$$\overline{3}$$ > pyramidal slip at room temperature.

**Figure 5:**
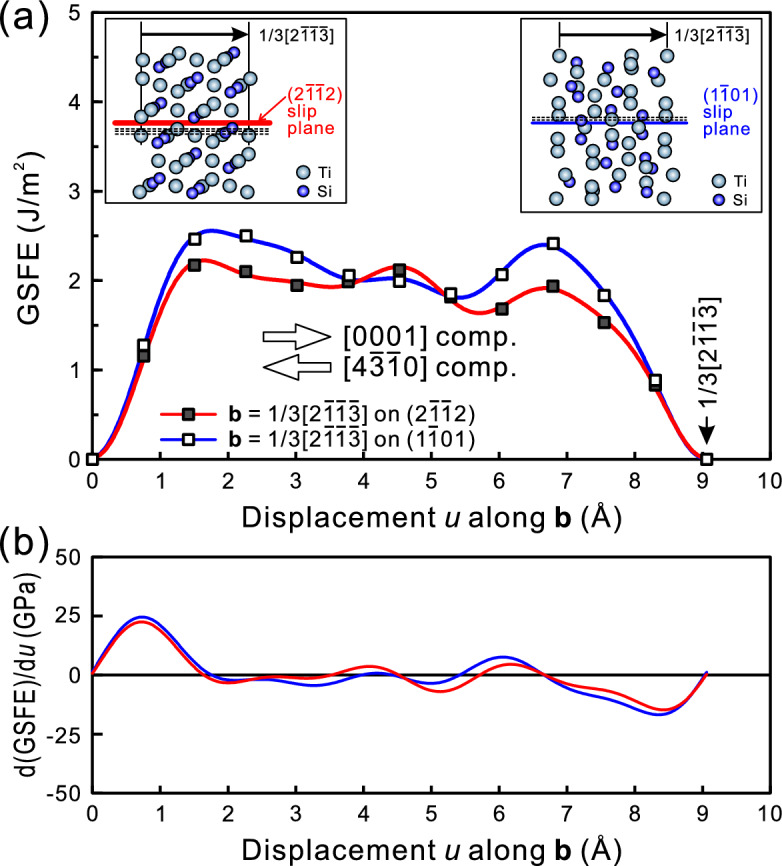
(**a**) Calculated GSFE curves and (**b**) their derivatives for two pyramidal slip systems of {2$$\overline{1}$$$$\overline{1}$$2} < 2$$\overline{1}$$$$\overline{1}$$$$\overline{3}$$ > and {1$$\overline{1}$$01} < 2$$\overline{1}$$$$\overline{1}$$$$\overline{3}$$ > .

For the [4$$\overline{3}$$$$\overline{1}$$0] orientation, on the other hand, while {2$$\overline{1}$$$$\overline{1}$$2} < 2$$\overline{1}$$$$\overline{1}$$$$\overline{3}$$ > pyramidal slip is observed in bulk single crystals at high temperatures, this slip was replaced by {1$$\overline{1}$$01} < 2$$\overline{1}$$$$\overline{1}$$$$\overline{3}$$ > pyramidal slip at room temperature for micropillar specimens. In order to see the relative ease in the operation for the slip along [2$$\overline{1}$$$$\overline{1}$$$$\overline{3}$$] (**a** + **c** slip) on two different pyramidal plane ((2$$\overline{1}$$$$\overline{1}$$2) and (1$$\overline{1}$$01)), generalized stacking fault energy (GSFE) for < 2$$\overline{1}$$$$\overline{1}$$$$\overline{3}$$ > slip on (2$$\overline{1}$$$$\overline{1}$$2) and (1$$\overline{1}$$01) were calculated by first-principles DFT calculations. Figure 5 plots the GSFE curves and their derivatives (the gradient of the GSFE curve corresponding to the ideal shear strength, τ_th_) for the two pyramidal slip systems of (2$$\overline{1}$$$$\overline{1}$$2)[2$$\overline{1}$$$$\overline{1}$$$$\overline{3}$$] and (1$$\overline{1}$$01)[2$$\overline{1}$$$$\overline{1}$$$$\overline{3}$$]. For (2$$\overline{1}$$$$\overline{1}$$2) and (1$$\overline{1}$$01) slip planes, there exist four and three different possible slip planes to be considered, respectively (see, the insets of Fig. 5a). Among the possible slip planes, only the calculation results for the slip planes exhibiting the lowest ideal shear strength and unstable stacking fault energies are indicated in Fig. 5. The selected slip planes are indicated with red and blue lines respectively, while the others are indicated with dashed black lines in the insets of Fig. 5a. To be noted in Fig. 5 is that the GSFE curves and their derivative curves are asymmetric on both slip planes with respect to the [2$$\overline{1}$$$$\overline{1}$$$$\overline{3}$$] slip direction and that the displacement along the positive direction gives rise to compression strain for the [0001] orientation while that along the negative direction does so for the [4$$\overline{3}$$$$\overline{1}$$0] orientation. Table 1 summarizes the results of the GSFE calculations. For both orientations, the ideal shear strength and unstable stacking fault energy are both lower on (2$$\overline{1}$$$$\overline{1}$$2) than on (1$$\overline{1}$$01), suggesting the preference for the (2$$\overline{1}$$$$\overline{1}$$2) slip plane for both orientations. This situation does not change even the ideal shear strength is normalized to the corresponding Schmid factors for each loading orientation (the normalized ideal shear strength, τ_th_/*m* in Table 1). The results of the GSFE calculations is consistent with what is observed in micropillar compression for the [0001] *orientation (the preference of the (2$$\overline{1}$$$$\overline{1}$$2) slip plane) but not with what is observed for the [4$$\overline{3}$$$$\overline{1}$$0] orientation. For the [4$$\overline{3}$$$$\overline{1}$$0] orientation, however, the normalized ideal shear strengths (τ_th_/*m*) on (2$$\overline{1}$$$$\overline{1}$$2) and (1$$\overline{1}$$01) slip planes differ from each other only by 3% (34.1 and 35.1 GPa, respectively). We believe that the difference in τ_th_/*m* is too small to draw a final conclusion for the slip plane preference for this orientation. One more thing to be noted in Table 1 is that the magnitude correlation for the ideal shear strength (τ_th_) for the (2$$\overline{1}$$$$\overline{1}$$2) and (1$$\overline{1}$$01) slip planes (22.5 and 16.8 GPa) is opposite to that for the bulk CRSS values deduced from micropillar experiment (1.90 and 2.32 GPa). We believe that this discrepancy comes from the fact that no dislocation dissociations, especially those accompanied by non-collinear Burgers vectors, are taken into account in the above discussion based on the GSFE calculations. In view of the large magnitude (about 0.906 nm) of the Burgers vectors of 1/3 < 2$$\overline{1}$$$$\overline{1}$$$$\overline{3}$$ > , the 1/3 < 2$$\overline{1}$$$$\overline{1}$$$$\overline{3}$$ > dislocation is expected to dissociate into partial dislocations with shorter Burgers vectors. In order to deduce the possible dissociation scheme for 1/3 < 2$$\overline{1}$$$$\overline{1}$$$$\overline{3}$$ > dislocation, construction of full gamma surfaces on (2$$\overline{1}$$$$\overline{1}$$2) and (1$$\overline{1}$$01) planes must be made, because experimental identification of the dissociation scheme is extremely difficult with the occurrence of instantaneous shear failure in micropillar specimens. The construction of gamma surface is currently in progress in the authors’ research group to draw the final conclusion on the slip plane preference.

**Table 1:** Summary of first-principles DFT calculations of generalized stacking fault energy (GSFE) for two pyramidal slip systems of {2$$\overline{1}$$$$\overline{1}$$2} < 2$$\overline{1}$$$$\overline{1}$$$$\overline{3}$$ > and {1$$\overline{1}$$01} < 2$$\overline{1}$$$$\overline{1}$$$$\overline{3}$$ > .

Compression axis	Slip direction	Slip plane	Schmid factor, *m*	Unstable stacking fault energy (J/m^2^)	Ideal shear strength, τ_th_ (GPa)	τ_th_/*m* (GPa)	Estimated bulk CRSS (GPa)
[4$$\overline{3}$$$$\overline{1}$$0]	[$$\overline{2}$$113] (negative direction)	(2$$\overline{1}$$$$\overline{1}$$2)	0.432	2.17	14.7	34.1	–
		**(1**${\overline{\mathbf{1}}}$**01)**	0.479	2.50	16.8	35.1	2.32
[0001]	[2$$\overline{1}$$$$\overline{1}$$$$\overline{3}$$] (positive direction)	**(2**$${\overline{\mathbf{1}}}$$$${\overline{\mathbf{1}}}$$**2)**	0.468	2.17	22.5	48.1	1.90
		(1$\overline{1}$01)	0.444	2.50	24.5	55.1	–

Slip planes indicated with bold letters correspond to those experimentally observed in this study.

## Methods

A single crystal rod of Ti_5_Si_3_ was grown from an ingot with a nominal composition of Ti − 37.5 at.% Si by directional solidification with an optical floating-zone furnace at a growth rate of 6 mm/h. Characteristics in the atomic arrangement and crystal structure of Ti_5_Si_3_ are detailed in our previous paper^19^. The crystal orientation of the grown single crystal was determined by the back-Laue X-ray diffraction method. Three loading-axis orientations, [2$$\overline{2}$$05], [4$$\overline{3}$$$$\overline{1}$$0] and [0001] were selected for micropillar compression tests. The highest Schmid factors for possible slip/twinning systems for each orientation are listed in Table 1 of our previous paper^19^. After the mirror finishing of specimen surface by mechanical polishing with diamond paste, micropillar specimens with a square cross-section having an edge length *L* ranging from 0.5 to 3.6 μm and a specimen height-to-*L* ratio of 1:3–1:5 were fabricated by the focused-ion beam (FIB) machining with a JEOL JIB-4000 FIB milling and imaging system operated at 30 kV. Micropillar compression tests were conducted at room temperature under the displacement-rate controlled mode (a nominal strain rate of 1 × 10^–4^ s^−1^) with an Agilent Technologies Nano Indenter G200 nanomechanical tester equipped with a flat-punch diamond indenter tip. Microstructures of micropillar specimens before and after the compression tests were investigated by scanning electron microscopy (SEM) in order to measure dimensions and analyze slip traces, respectively. SEM observations were made along the direction inclined by 30° from the loading axis. Single cantilever bend tests were conducted with chevron-notched micro-beam specimens to evaluate the fracture toughness, as the details of the experimental procedures are described in our previous paper^24^.

Generalized stacking fault energy (GSFE) for 1/3 < 2$$\overline{1}$$$$\overline{1}$$$$\overline{3}$$ > slip on (2$$\overline{1}$$$$\overline{1}$$2) and (1$$\overline{1}$$01) were calculated by first-principles DFT calculations using the Vienna ab-initio simulation package (VASP) code^45–47^. The generalized gradient approximation of Perdew–Burke–Ernzerhof (GGA-PBE) was used to treat the exchange–correlation functional^48^. We used supercells for the GSFE calculations for (2$$\overline{1}$$$$\overline{1}$$2) and (1$$\overline{1}$$01) planes respectively containing 192 and 96 atoms, in each of which the Ti:Si atomic ratio was fixed at 5:3 so as to maintain the stoichiometry of Ti_5_Si_3_. The in-plane units of the supercells were defined by unit vectors along [2$$\overline{1}$$$$\overline{1}$$$$\overline{3}$$] and [01$$\overline{1}$$0] for the (2$$\overline{1}$$$$\overline{1}$$2) supercell and by those along [2$$\overline{1}$$$$\overline{1}$$$$\overline{3}$$] and [1$$\overline{2}$$13] for the (1$$\overline{1}$$01) supercell. A vacuum layer with a total thickness of 1.5 nm (when measured along the slip plane normal) is included in each supercell. Monkhorst–Pack *k*-point meshes of 10 × 8 × 2 and 8 × 8 × 2 were used for the (2$$\overline{1}$$$$\overline{1}$$2) and (1$$\overline{1}$$01) supercells, respectively^49^. An energy cutoff of 500 eV was used throughout the calculations. All atoms were relaxed only along the direction perpendicular to the glide plane so as to minimize the energy of the supercell with a given in-plane displacement^24^.

## Conclusions

The room-temperature deformation behavior of Ti_5_Si_3_ was investigated by micropillar compression tests as a function of loading-axis orientation and specimen size and micro-cantilever bend tests with chevron-notched specimens. The results obtained are summarized as follows.

1. Three slip systems, {1$$\overline{1}$$00}[0001], {2$$\overline{1}$$$$\overline{1}$$2} < 2$$\overline{1}$$$$\overline{1}$$$$\overline{3}$$ > and {1$$\overline{1}$$01} < 2$$\overline{1}$$$$\overline{1}$$$$\overline{3}$$ > , are operative at room temperature. The CRSS values for all three slip systems increase with the decrease in specimen size in the range of 2.7–6 GPa, following an inverse power-law relationship with the power-law exponents of 0.36, 0.30 and 0.11 for {1$$\overline{1}$$00}[0001], {2$$\overline{1}$$$$\overline{1}$$2} < 2$$\overline{1}$$$$\overline{1}$$$$\overline{3}$$ > and {1$$\overline{1}$$01} < 2$$\overline{1}$$$$\overline{1}$$$$\overline{3}$$ > slip, respectively. The bulk CRSS values for these three slip systems are estimated to be 1.43 ± 0.10, 1.90 ± 0.10 and, 2.32 ± 0.06 GPa by extrapolating the inverse power-low curves to the specimen size range of 20–30 μm.
2. The fracture toughness values are evaluated by micro-cantilever bend tests to be 0.45 and 0.73 MPa m^1/2^ with chevron-notch planes parallel to (0001) and (1$\overline{1}$00), respectively. The fracture toughness values are considerably lower than those for D8_*l*_-Mo_5_SiB_2_ (2.43 MPa m^1/2^; notch plane // (100)) and D8_*l*_-Nb_5_Si_3_ (1.79 MPa m^1/2^; notch plane // (001)) evaluated by the same method, indicating much more significant inherent brittleness of Ti_5_Si_3_ compared to the other transition-metal silicides of the TM_5_Si_3_ type.

## Data Availability

The datasets generated during and/or analysed during the current study are available from the corresponding author on reasonable request.
